# Gripping Prospective of Non-Shear Flows under High-Pressure Torsion

**DOI:** 10.3390/ma16020823

**Published:** 2023-01-14

**Authors:** Yan Beygelzimer, Yuri Estrin, Oleksandr Davydenko, Roman Kulagin

**Affiliations:** 1 Institute of Nanotechnology, Karlsruhe Institute of Technology, Hermann-von-Helmholtz-Platz 1, 76344 Eggenstein-Leopoldshafen, Germany; 2 Donetsk Institute for Physics and Engineering Named after O.O. Galkin, National Academy of Sciences of Ukraine, Nauky Ave., 46, 03028 Kyiv, Ukraine; 3 Department of Materials Science and Engineering, Monash University, 22 Alliance Lane, Clayton, VIC 3800, Australia; 4 Department of Mechanical Engineering, The University of Western Australia, 35 Stirling Highway, Perth, WA 6009, Australia

**Keywords:** high-pressure torsion, slippage, plastic deformation, stress, strain

## Abstract

The article presents a theoretical study of the regimes of high-pressure torsion (HPT) for which slippage of the deforming material on the interfaces with anvils is possible. The approach taken is a generalisation of the currently accepted view of the HPT process. It enables a rational explanation of its salient features and the effects observed experimentally. These include a lag in the rotation angle of the specimen behind that of the anvils, an outflow of the material from the deformation zone, enhancement in gripping the specimen with anvils with increasing axial pressure, etc. A generalised condition for gripping the specimen with anvils, providing a basis for an analytical investigation of the HPT deformation at a qualitative level, is established. The results of the analytical modelling are supported by finite-element calculations. It is shown that for friction stress below the shear stress of the specimen material (i.e., for the friction factor m < 1), plastic deformation is furnished by non-shear flows, which expands the range of possible process regimes. The potential of these flow modes is impressive, which is reflected in the second meaning of the word “gripping” in the title of the article. Non-shear flows manifest themselves in the spreading of the material over the anvil surfaces whose cessation signifies the end of deformation and the beginning of slippage of the specimen as a whole. The model shows that for m < 1 such a finale is inevitable at any axial pressure. It predicts, however, that the highest achievable strain is increased when the axial pressure is raised in the course of the HPT process. Unlimited deformation of the specimen is only possible for m = 1, when slippage of the deforming material relative to the anvils is suppressed.

## 1. Introduction

Despite its seeming simplicity, high-pressure torsion (HPT) is remarkably efficient. It was proposed in the 1930s by the Nobel Prize winner P. Bridgman as a way to induce phase transformations and chemical reactions under high pressure [1,2]. Since the 1990s, HPT has been used as the most popular method for obtaining ultrafine-grained metals and alloys via severe plastic deformation [3,4,5]. Over the last 10 years the method has been applied for fabrication of new materials with different compositions whose constituents react with each other during co-deformation under high pressure [6,7,8,9,10,11].

The realization of HPT involves seizure of the specimen by anvils, which, through the contact surfaces, impart to it a torque. Ensuring reliable seizure is key to any application of this process. At present, the knowledge of how seizure occurs and what influences it is rather incomplete, however. What is known can be summarised as follows.

If seizure is insufficient, slippage of the specimen relative to the anvils occurs. This reduces the achievable plastic strain, which in extreme cases can vanish altogether [12,13,14]. Experiments show a clear tendency to increased slippage with increasing hardness of the specimen material. For hard materials such as iron or steel, slippage intensifies with increasing angular velocity of the revolving anvils [12]. To enhance seizure, one applies high axial pressure and uses anvils treated by sanding.

Starting from the seminal publications by P. Bridgman [1,15], it is commonly agreed that seizure of a specimen by anvils is a result of friction stress $\tau_{fr}$ on the contact surfaces reaching the magnitude of the shear stress k of the deforming material:

(1) $$\tau_{fr}=k,$$

Equation (1) means that any relative displacements of two contacting bodies at the interface are blocked while external friction is transformed to plastic shear of the softer of the two materials [15,16,17]. Accordingly, in mathematical modelling of HPT, the boundary conditions at the surfaces in contact with each other are set either in the form of Equation (1) or through the sticking condition [18,19,20,21].

The seizure, or gripping, criterion, expressed by Equation (1), raises some questions about contact friction. The friction stress at high normal pressure is determined by the following equation:

(2) $$\tau_{fr}=mk,$$

Here, 0<m≤1 is the friction factor, which is equal to unity only for an ideally clean juvenile surface of a specimen. In the presence of surface films, such as contaminated layers or oxides, the inequality m<1 holds [16,17]. How, then, can HPT be realised if, as a rule, the surfaces of specimens are covered with such films? The gripping condition, Equation (1), is valid if HPT is viewed as a process of plastic shear on a plane parallel to the anvil work surface [1,15]. However, in reality this is not always the case, as was mentioned by P. Bridgman, who wrote, “The actual distribution of stress and strain in the disk is evidently very complicated, and must differ greatly from the mean values…” [1]. Recent studies confirm this remark [18,19,20,21,22] and question the necessity of Equation (1) to be satisfied. In particular, it was shown by computations using the commercial rigid/plastic finite element code DEFORM that gripping of a specimen is also possible for m<1 [22]. In that case, a decrease in the friction factor reduces the maximum effective strain attainable by HPT for a given pressing force on the anvils.

In this article we propose an extended interpretation of the HPT process, in which non-shear flows of the specimen material are allowed. This makes it possible to obtain a generalised gripping condition, Equation (1), being a special case thereof. We use this generalised condition to provide a rational explanation for several HPT effects associated with specimen gripping. The reader is reminded again that we use the term “gripping” as being synonymous with “seizure”, which is commonly used in the literature on friction.

## 2. Generalised Gripping Conditions

We demonstrate below that the gripping condition expressed by Equation (1) is valid only if non-shear flows are blocked. If they are admitted, plastic deformation of the specimen under HPT is also enabled for $\tau_{fr}<k$. To that end, we consider the von Mises plasticity criterion, which reads [23]:

(3) $${\left(\sigma_{zz}-\sigma_{rr}\right)}^{2}+{\left(\sigma_{zz}-\sigma_{\theta \theta }\right)}^{2}+{\left(\sigma_{\theta \theta }-\sigma_{rr}\right)}^{2}+6\left(\sigma_{zr}^{2}+\sigma_{z\theta }^{2}+\sigma_{\theta r}^{2}\right)=6k^{2}$$

**Figure 1:**
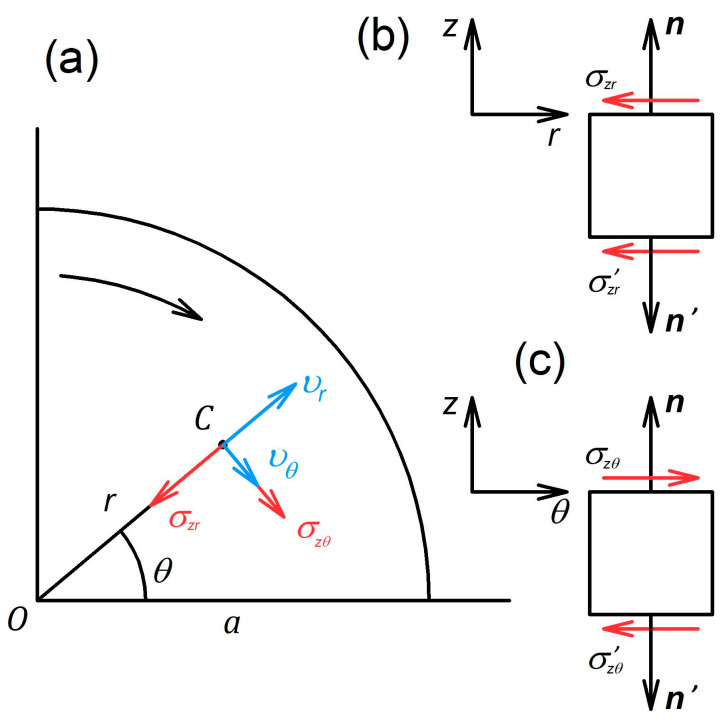
Schematics explaining the directions of the shear stresses on the contact surfaces between the specimen and the anvils: (**a**) upper surface normal to the anvil rotation axis, (**b**,**c**) cross-sections of the specimen perpendicular to the θ- and r-axes, respectively. The rotation of the upper anvil is indicated by the curved arrow. The notation in the figure is as follows: $v_{r}\;\mathrm{and}\;v_{\theta }$ are the components of the velocity vector at point *C*; n and $n^{\prime }$ are the vectors normal to the upper and lower surfaces of the specimen, respectively. All other symbols are defined in the text.

Here, $\sigma_{ij}$ denotes the components of the stress tensor in cylindrical coordinates with the *z*-axis directed opposite to the force direction in Figure 1.

We set $\sigma_{rr}=\sigma_{\theta \theta }$—an assumption common for the case of axial symmetry [23,24]. In addition, we assume that shear occurs mainly in the direction normal to the *z*-axis, i.e., that the inequalities $\left|{\dot{\gamma }}_{\theta r}\right|\ll \left|{\dot{\gamma }}_{zr}\right|$ and $\left|{\dot{\gamma }}_{\theta r}\right|\ll \left|{\dot{\gamma }}_{z\theta }\right|$ hold. It then follows from the Associated Flow Law [23] that $\left|\sigma_{\theta r}\right|\ll \left|\sigma_{zr}\right|$ and $\left|\sigma_{\theta r}\right|\ll \left|\sigma_{z\theta }\right|$, which allows us to use the equation $\sigma_{\theta r}=0$. Using these assumptions in Equation (3), we obtain the following plasticity condition:

(4) $${\left(\sigma_{zz}-\sigma_{rr}\right)}^{2}+3\tau^{2}=3k^{2},$$

where

(5) $$\tau =\sqrt{\sigma_{zr}^{2}+\sigma_{z\theta }^{2}},$$

is the shear stress in the plane normal to the *z*-axis.

According to the Associated Flow Law, the strain rates for this case obey the following relations:

(6) $$\dot{\gamma }=\lambda \frac{\partial f}{\partial \tau },\;\;{\dot{e}}_{zz}=\lambda \frac{\partial f}{\partial \sigma_{zz}},\;\;{\dot{e}}_{rr}=\lambda \frac{\partial f}{\partial \sigma_{rr}},$$

with

(7) $$\dot{\gamma }=\sqrt{{\dot{\gamma }}_{zr}^{\;2}+{\dot{\gamma }}_{z\theta }^{\;2}},$$

and

$$f={\left(\sigma_{zz}-\sigma_{rr}\right)}^{2}+3\tau^{2}-3k^{2},$$

Here, λ is a parameter [23] that does not enter the subsequent analysis.

In fully constrained HPT [3], when the specimen is placed in a cavity within an anvil whose walls block the outflow of the material, one has ${\dot{e}}_{rr}\equiv 0$. It then follows from Equation (6) that $\frac{\partial f}{\partial \sigma_{rr}}\equiv$ 0, which is only possible if $\sigma_{zz}\equiv \sigma_{rr}$ holds. In this case, the plasticity condition, Equation (3), takes the following form:

(8) τ=k,

Further conditions satisfied for fully constrained HPT read as follows: ${\dot{\gamma }}_{zr}=0$ and $\sigma_{zr}=0$ [20]. Combining Equations (5), (7), and (8), one than obtains $\dot{\gamma }=\left|{\dot{\gamma }}_{z\theta }\right|$ and $\tau =\left|\sigma_{z\theta }\right|$. At the contact surfaces the equality $\sigma_{z\theta }=\tau_{fr}$ is fulfilled. Assuming the uniformity of $\sigma_{z\theta }$ throughout the specimen thickness, we find from Equation (8) that $\tau_{fr}=k$ holds. That is to say, Equation (1) is a necessary condition for plastic deformation of the specimen under full-constraint HPT when non-shear flow is blocked by the walls of the anvil cavity. The corresponding sufficient conditions were obtained in [20].

We now turn to the question of how non-shear flows affect the gripping of a specimen. First, we need to formally define the notion of gripping. We shall consider the specimen to be gripped by anvils if the torque and the pressure applied to it cause plastic deformation. This definition is broader than the currently accepted one, which implies sticking the specimen to the anvils. Indeed, our definition does not rule out the possibility of slippage. The latter must take place if the material is pressed out of the working zone of the anvils. We show below that this extended interpretation of gripping helps obtain the full picture, which includes both shear and non-shear flows, thus answering the questions posed in the introduction.

To obtain a gripping criterion based on the new definition and generalising Equation (1), we consider the magnitude of τ. Figure 1 displays a schematic illustrating the direction of shear stresses on the flat specimen surfaces. In a general case, it is assumed that the specimen is twisted and compressed by the anvils. As a result of axial compression, the specimen material is pressed out in radial directions. Any point on the specimen surface, except the ones located on the rotation axis, has non-zero components of the velocity along the r- and θ-axes.

The components of the vector of shear stress on the specimen surface are nothing else but the components of the friction stress developing between the specimen and the anvils. Friction is passive in radial directions since the anvil hinders radial outflow of the material. The stress $\sigma_{zr}$ thus acts against $v_{r}$. In the tangential direction, friction is active, as the specimen is twisted by the anvils. Hence, $\sigma_{z\theta }$ acts in the direction of $v_{\theta }$. As follows from Figure 1, $\sigma_{z\theta }\;$ has the same orientation with respect to the normal vector, i.e., it has the same sign on the upper and the lower specimen surfaces, whereas $\sigma_{zr}$ has the opposite signs on the two surfaces.

The abovementioned leads to the conclusion that, in a first approximation, the following relations hold for the specimen bulk: $\sigma_{z\theta }={\left(\tau_{fr}\right)}_{\theta }$ and $\sigma_{zr}=\left(2z/h\right){\left(\tau_{fr}\right)}_{r}$, where ${\left(\tau_{fr}\right)}_{\theta }$ and ${\left(\tau_{fr}\right)}_{r}$ are, respectively, the tangential and the radial components of the friction stress; h denotes the specimen thickness. Substitution of these relations in Equation (5) yields

(9) $$\tau =\sqrt{{\left[\left(2z/h\right){\left(\tau_{fr}\right)}_{r}\right]}^{2}+{\left[{\left(\tau_{fr}\right)}_{\theta }\right]}^{2}}$$

This function has a minimum in the mid-section of the specimen, at z=0,  and reaches the maximum value, $\tau_{max}=\tau_{fr}$, at the specimen surfaces at z=±h/2. This means that the magnitude of the friction stress limits the possible values of τ. With the account of Equation (2), we record this conclusion in the inequality ≤mk, which is to be fulfilled in conjunction with the plasticity condition, Equation (4). Hence, the criterion, which by necessity must be satisfied by the stresses within the specimen during its plastic deformation imparted to it by the anvils, reads:

(10) $$\left\{\begin{array}{c} {\left(\sigma_{zz}-\sigma_{rr}\right)}^{2}+3\tau^{2}=3k^{2}\\ \tau \leq mk \end{array}\right.$$

Criterion (10) represents the gripping criterion corresponding to the above generalised notion.

In the absence of non-shear flows when $\sigma_{zz}=\sigma_{rr}$ holds, Criterion (10) is reduced to:

(11) $$\left\{\begin{array}{c} \tau =k\\ \tau \leq mk \end{array}\right.$$

From this, the equality m=1 follows. Hence, for the special case of shear flow, the generalised gripping criterion recovers the known criterion given by Equation (1).

The result thus obtained can be elucidated graphically. For this purpose, we transform plasticity condition of Equation (4) to the following form:

$${\left(\sigma_{zz}-\sigma \right)}^{2}+{\left(\frac{\sqrt{3}}{2}\tau \right)}^{2}={\left(\frac{\sqrt{3}}{2}k\right)}^{2}$$

where $\sigma =\frac{\sigma_{zz}+\sigma_{rr}}{2}$.

**Figure 2:**
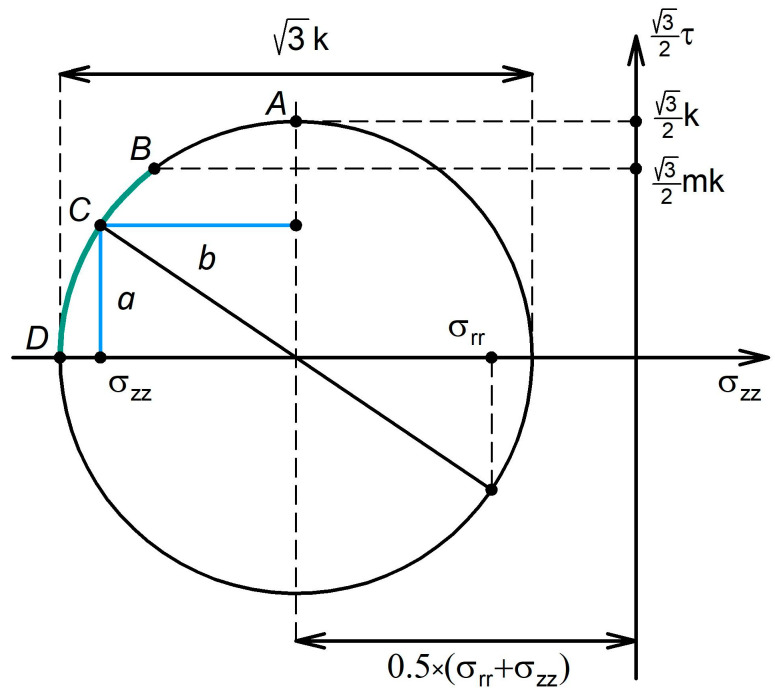
Graphical representation of the gripping condition for an HPT specimen.

It follows from the previous condition that in coordinates $\left(\sigma_{zz},\frac{\sqrt{3}}{2}\tau \;\right)$ Equation (4) is the equation of a circle, with a radius of $\frac{\sqrt{3}}{2}k$ and the centre at the point $\left(\sigma ,0\right)$. The corresponding graph is shown in Figure 2.

The plasticity condition, Equation (4), is represented by the circle in Figure 2. At its apex (point A), the relation τ=mk holds. According to Criterion (10), the stress state of a plastically deforming specimen is mapped to the points on the arch BD, which we refer to as the “gripping arch.” Based on the Associated Flow Law, Equation (6), for any point C on the circle, the length of the segments *a* and *b* are related to the deformation rates $\dot{\gamma }$ and ${\dot{e}}_{zz}$ through the following equations:

(12) a=γ˙43λ

(13) b=e˙zz4λ

As seen from Figure 2, the shear stress is non-zero on the entire arch AD, apart from its apex A, where b=0 holds. It follows from Equation (13) that only at point A, i.e., for τ=k, is the deformation of the specimen not accompanied by non-shear flows (${\dot{e}}_{zz}=0)$. Accordingly, gripping of the specimen for m<1 must necessarily involve non-shear flows (${\dot{e}}_{zz}\neq 0,{\dot{e}}_{rr}\neq 0$), expressed through extrusion of the material out of the working zone of the anvils.

## 3. Plastic Flow of an HPT Specimen under Generalised Gripping Condition

Commonly, non-shear flows are presented as a passive factor, contrary to shear flow, which is considered active. This viewpoint is also common in assessments of the role of the force elements of HPT rigs, which include two independent drivetrains: one producing the axial force P and the other the torque *M*. The conventional view is that the axial force is used to induce high pressure and ensure seizure of the specimen by the anvils while the plastic deformation is effected by the torque. However, in reality both drivetrains produce the work of deformation when non-shear flows occur. We take an alternative standpoint and posit that for efficient control of material flows under HPT both force factors are to be considered on equal terms. Below, this idea is outlined by considering generalised variables, which characterise the state of the system [24,25].

The expression for the power of the external forces, $\dot{W}$, is written as follows:

(14) $$\dot{W}=M\Omega +PU$$

Here Ω and U denote the relative angular rotation velocity and the relative translational displacement speed of the anvils, respectively.

In light of Equation (14), the quantities *M* and *P* can be regarded as generalised forces and Ω and U as generalised velocities related to *M* and *P* through the following equations:

(15) $$\Omega =\Lambda \frac{\partial F}{\partial M}$$

(16) $$U=\Lambda \frac{\partial F}{\partial P}$$

Here $F=F\left(M,P\right)$ is the plastic potential, which defines the generalised plasticity condition: The specimen deforms plastically if $F\left(M,P\right)=0$ holds, whereas it is in the elastic state under the condition that $F\left(M,P\right)<0$ [24,25].

Let us derive an expression for the plastic potential for the HPT deformation. According to [23], the mean pressure $p_{0}$ required for the plastic compression of a thin round disk is given by the following formula:

(17) $$p_{0}=\frac{kR}{3h}$$

where 2R and 2h are, respectively, the diameter and the thickness of the disk.

This expression was obtained for sufficiently thin specimens when $\left(h/R\right)\ll 1$ holds and assuming that there is no obstruction to its outflow at the disk edges (unconstrained HPT). This condition can be generalised by considering flow inhibition under quasi-constrained HPT. The hindrance to the outflow can be represented by some radial pressure q applied at the disk perimeter. It is caused by extrusion of the specimen material into a ring-shaped gap between the anvils from the cavity housing the specimen [3].

To determine how this radial pressure will alter Equation (17), we conduct a thought exercise in which a hydrostatic pressure q is applied to the disk in addition to the axial pressure. Neither the plasticity condition nor the limiting friction will be affected by that. This means that the new stress state will satisfy the plasticity conditions [23], and the required radial pressure q  at the disk perimeter will be imposed. The mean the axial stress, previously provided by Equation (17), will be raised by the same amount:

(18) $$p_{0}=\frac{kR}{3h}+q$$

The expression for the corresponding axial force $P_{0}=\pi R^{2}p_{0}$, which is necessary for compression of the disk in the absence of a torque, then reads:

(19) $$P_{0}=\frac{k\pi R^{3}}{3h}+Q$$

with $Q=\pi R^{2}q$.

Let us now consider the opposite extreme: plastic torsion of a round disk “welded” to the anvils (the case of limiting friction regardless of the applied pressure). The torque required for that is given [24] by

(20) $$M_{0}=\frac{2}{3}k\pi R^{3}$$

Under Conditions (19) and (20) non-shear flows and the shear flow are realised in their pure forms. If the inequalities $P<\frac{k\pi R^{3}}{3h}+Q$ and $M<\frac{2}{3}k\pi R^{3}$ hold, plastic deformation is induced by two drivetrains and the material flow has two components. In this case, the axial force and the torque must be related through a generalised plasticity condition, like the components of the stress tensor expressed in terms of the plasticity condition of Equation (3).

We assume that the generalised plasticity condition is a quadratic function of M and P [24,25] satisfying Equations (19) and (20) and write this condition as

(21) $${\left[2h\left(P-Q\right)\right]}^{2}+M^{2}=K^{2}$$

where $K=\frac{2}{3}k\pi R^{3}$.

**Figure 3:**
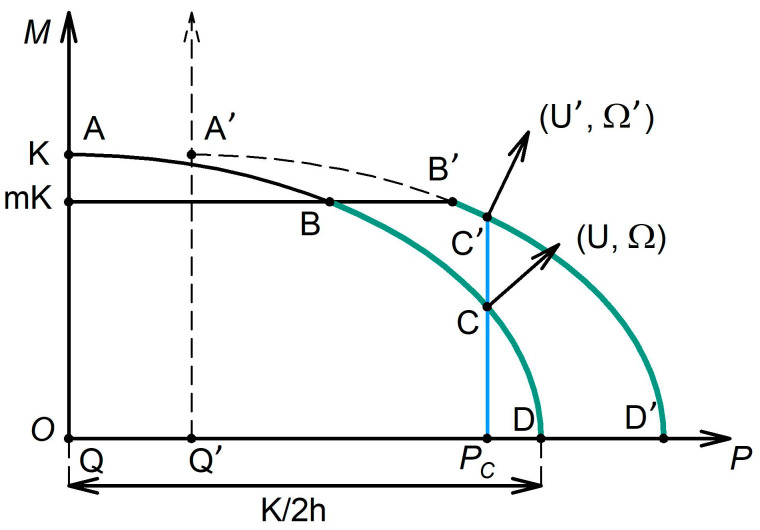
Generalised plasticity locus for HPT. The range where the HPT process is possible is marked in green.

Equation (21) describes an ellipse with the half-axes K/2h and K in the coordinate system (P,M) shifted upwards along the P-axis by an amount Q. In Figure 3, the arch AD mapping the generalised plasticity locus is shown.

According to the above definition of the plastic potential (cf. Equations (15) and (16), as well as the text immediately below them), the expression for *F* that follows from Equation (21) reads

(22) $$F={\left[2h\left(P-Q\right)\right]}^{2}+M^{2}-K^{2}$$

Substitution of Equation (22) into Equations (15) and (16) yields the following expressions for the generalised velocities:

(23) Ω=2M

(24) $$U=8\partial h^{2}\left(P-Q\right)$$

According to Equations (15) and (16), the generalised velocity vector $\left(U,\Omega \right)$ is normal to the plasticity locus at any point on it, cf. Figure 3.

All regimes on the arch AD can be realised only for the limiting friction between the specimen and the anvils, i.e., for m=1. For m<1, when the contact zone is contaminated by impurities or oxides, the greatest possible torque has the magnitude of $M_{B}$, where

(25) $$M_{B}=\frac{2}{3}mk\pi R^{3}=mK$$

If, as determined by Equation (21), the plastic deformation requires a higher value of the torque, slippage of the specimen relative to the anvils will occur, as friction cannot deliver the necessary torque level. This indicates that plastic deformation only happens on a part of the arch, between *D* and *B* (see Figure 3). We refer to this part of the arch as the HPT feasibility region. In a certain sense, it corresponds to the “gripping arch” presented in Figure 2.

It can now be demonstrated that with the progress of straining, the HPT regime with a constant axial force is shifted towards the boundary of the HPT feasibility region *B*. Let a point C represent the plastic deformation condition at a certain moment. Since at this point U  is non-zero, the thickness h of the specimen will decrease with the growing anvil rotation angle, as U is the relative translational displacement speed of the anvils. As a result, the half-axis of the plasticity ellipse K/2h will increase. In addition, due to the narrowing of the gap between the anvils upon their translational motion, the radial pressure q will go up. Consequently, Q will grow to a certain value $Q^{\prime }$, leading to a shift of the plasticity ellipse to the right. The generalised plasticity locus corresponding to $h^{\prime }<h$ is shown as a dotted line in Figure 3. (For simplicity, K=const is used.) For a constant axial force (indicated as $P_{c}=const$ in Figure 3), an increase in the anvil rotation angle will lead to an increase in the ordinate of point *C*, which moves closer to the boundary of the HPT feasibility region. As seen from Figure 3, the magnitude of U will then drop, which signifies a decrease in the intensity of the material outflow from the working zone of the anvils. Once the representative point on the plasticity arch has reached the boundary of the HPT feasibility region, plastic deformation will cease and the entire specimen will be slipping relative to the anvils.

Let us outline the path to obtaining quantitative results for HPT deformation by using the generalised plasticity-locus approach.

Since Ω and U denote the relative angular rotation velocity and the relative translational displacement speed of the anvils, one has Ω=dφ/dt and U=−2dh/dt, where t is the process time and φ is the relative anvil rotation angle. Using these relations and combining Equations (23) and (24), we arrive at the following differential equation for h:

(26) $$\frac{dh}{d\varphi }=-\frac{2h^{2}\left(P-Q\right)}{M}$$

Now assume that Q=CP, where 0≤C≤1. For the limit cases of non-constrained and fully constrained HPT, the equalities C=0 and C=1 hold, respectively. Intermediate values of C correspond to quasi-constrained HPT with various degrees of hindrance to the plastic flow of the metal. By substituting the value of M found from the plasticity condition of Equation (21) into Equation (26) and employing the above expression for Q,  we obtain

(27) $$\frac{dh}{d\varphi }=-\frac{2\left(1-C\right)Ph^{2}}{\sqrt{K^{2}-4{\left(1-C\right)}^{2}P^{2}h^{2}}}$$

Expressing the axial force in terms of the average pressure $\left(P=\pi R^{2}p\right)$ and introducing non-dimensional variables $\bar{h}=h/R$, $\bar{M}=M/K$, $\bar{p}=p/\sigma_{s}$ ($\mathrm{where}\;\sigma_{s}=\sqrt{3}k$ is the flow stress of the deforming material), we arrive at the following differential equation:

(28) $$\frac{d\bar{h}}{d\varphi }=-\frac{3\sqrt{3}\left(1-C\right)\bar{p}{\bar{h}}^{2}}{\sqrt{1-27{\left(1-C\right)}^{2}{\bar{p}}^{2}{\bar{h}}^{2}}}$$

In non-dimensional variables, the highest attainable torque given by Equation (25) assumes the form

(29) $${\bar{M}}_{B}=\sqrt{1-27{\left(1-C\right)}^{2}{\bar{p}}^{2}{\bar{h}}^{2}}\leq m$$

Equation (28), taken together with the limiting condition of Equation (29), enables the calculation of $\bar{h}\left(\varphi \right)$ for a prescribed loading schedule $\bar{p}\left(\varphi \right)$ and the initial condition $\bar{h}\left(0\right)={\bar{h}}_{0}$.

Let us now obtain a relation for assessing the equivalent, or effective, von Mises strain $e_{eff}$, which accounts for non-shear flow. According to [23], we have

(30) $$e_{eff}=\int_{0}^{t}{\dot{e}}_{eff}dt$$

with

(31) e˙eff=23e˙zz−e˙rr2+e˙zz−e˙θθ2+e˙rr−e˙θθ2+32γ˙zr 2+γ˙zθ 2+γ˙rθ 2

Considering the assumptions introduced in Section 1, Equation (31) reduces to

(32) e˙eff=γ˙31+3e˙zzγ˙2

where $\dot{\gamma }$ is given by Equation (7). The quantities entering this relation are taken as

(33) $$\dot{\gamma }=\frac{\left|v_{\theta }\left(r\right)\right|}{h}$$

(34) e˙zzγ˙=1hdhdφ

Substitution in Equation (32) yields

(35) $${\dot{e}}_{eff}\left(r\right)=\frac{1}{\sqrt{3}}\sqrt{1+3{\left(\frac{1}{h}\frac{dh}{d\varphi }\right)}^{2}}\frac{\left|v_{\theta }\left(r\right)\right|}{h}$$

where r is the distance from the rotation axis.

As the work produced by the torque is positive, friction is active in the direction of θ, and $\left|v_{\theta }\left(r\right)\right|<r\Omega$ holds. Therefore, it follows for an upper estimate of ${\dot{e}}_{eff}$ that

(36) $${\dot{e}}_{eff}\left(r\right)=\frac{1}{\sqrt{3}}\sqrt{1+3{\left(\frac{1}{h}\frac{dh}{d\varphi }\right)}^{2}}\frac{r}{h}\frac{d\varphi }{dt}$$

Substituting this relation in Equation (30) and using non-dimensional variables, we obtain the sought estimate of the effective von Mises strain, which accounts for non-shear flows:

(37) $$e_{eff}\left(\bar{r}\right)=\frac{\bar{r}}{\sqrt{3}}\int_{0}^{\varphi }\sqrt{1+3{\left(\frac{1}{\bar{h}}\frac{d\bar{h}}{d\varphi }\right)}^{2}}\frac{d\varphi }{\bar{h}}$$

where $\bar{r}=r/R$ and $\bar{h\left(\varphi \right)}$ are determined by Equation (28) combined with the condition of Equation (29).

**Figure 4:**
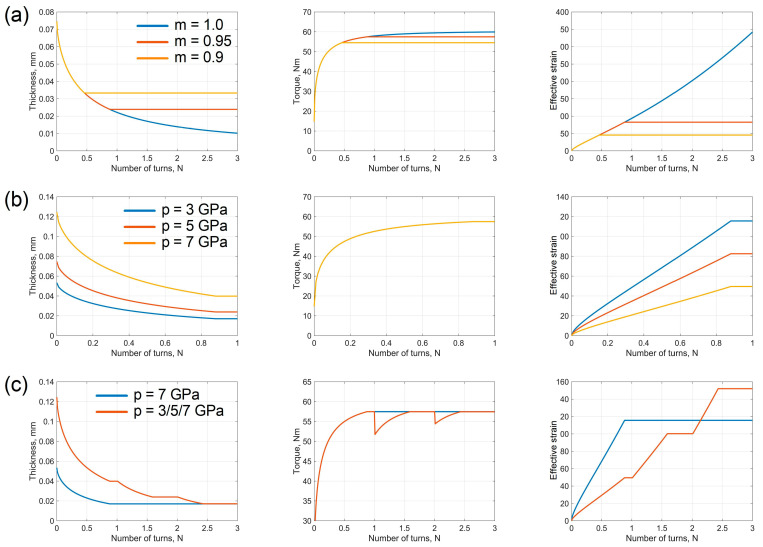
The dependence of the specimen thickness, torque, and effective strain on the number of anvil revolutions according to the model presented (**a**) at a fixed axial pressure of 5 GPa; (**b**) at a fixed friction factor of m =0.95; (**c**) the effect of the loading schedule.

The above mathematical model of HPT based on the generalised gripping condition predicts several effects, illustrated in Figure 4. The following numerical values of the quantities involved were used in the calculations of $h=0.5\;\mathrm{mm},\;R=5\;\mathrm{mm},\;\;\sigma_{s}=400\;\mathrm{MPa}$, and non-constrained HPT conditions (C=0).

According to our model, deformation under non-constrained (C=0) and quasi-constrained (0<C<1) HPT is accompanied by a reduction in the specimen thickness and its concomitant spreading over the anvil surfaces. If the friction is below the limit (m<1), then after a certain amount of anvil rotation slippage of the specimen as a whole sets in and no deformation occurs. The higher the friction factor (Figure 4a) and the axial pressure (Figure 4b), the greater the strain accumulated up to that moment. The magnitude of the effective strain is also influenced by the schedule according to which the axial pressure is applied. The blue line corresponds to the applied axial pressure of 7 GPa, with a subsequent anvil rotation; the red line refers to incremental increase in the pressure from 3 GPa to 7 GPa in 2 GPa steps (each step terminating with the onset of slippage of the specimen). For the same final pressure, the maximum attainable effective strain is increased if the final pressure is not fixed from the start of loading but rather is increased to its highest level in a staggered way as the anvil rotation is increased (Figure 4c).

## 4. Numerical Analysis of Gripping

In the foregoing sections gripping of the specimen was investigated qualitatively using, where possible, an analytical approach. Below we present the results of a quantitative numerical study of the HPT process in different regimes.

### 4.1. Computation Methodology

**Figure 5:**
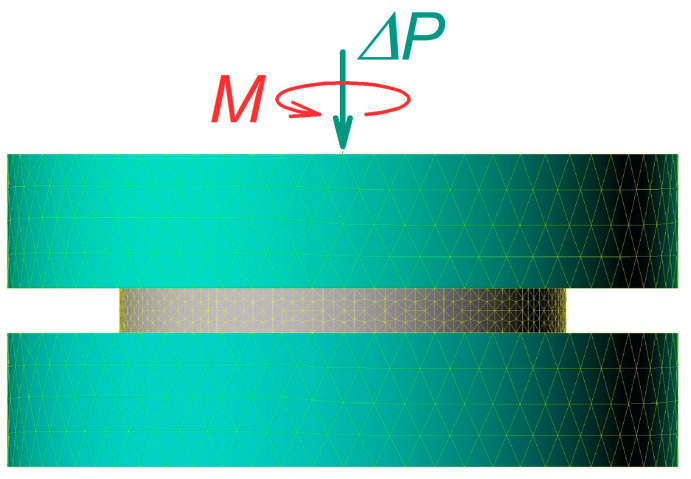
Geometrical model used in computations of the HPT process.

Computations were carried out using the commercially available finite-element package QForm-3D [26]. A geometrical sketch of the system is shown in Figure 5.

It is assumed that the lower anvil is fixed, whereas the upper one is rotated at a constant angular velocity Ω=2 rpm. A force aligned with the rotation axis is applied at the upper anvil. The specimen is disk-shaped and has an initial thickness of 1 mm and a diameter of 10 mm. The specimen’s material parameters are those of pure copper. The friction stress at the anvil–specimen interfaces is given by Equation (2). It is further assumed that a sufficiently high hydrostatic pressure (three- to four-fold that of the flow stress of the material) is applied to the system. Despite having no effect on the equations of the plasticity theory, this pressure brings the friction stress to the level when Equation (2) is valid. This makes it possible to study the behaviour of the system on the arch AD of the Mohr circle by varying the friction factor  m without bothering with its location on the abscissa, which is defined by the magnitude of the hydrostatic stress. Thus, the force ΔP is determined by the (positive) difference between $\left|\sigma_{zz}\right|$ and the hydrostatic pressure, rather than the magnitude of $\left|\sigma_{zz}\right|$.

Two sets of numerical calculations were carried out. In the first one, the initial stage of gripping was modelled. In the second, its development under a constant force ΔP was modelled.

**Figure 6:**
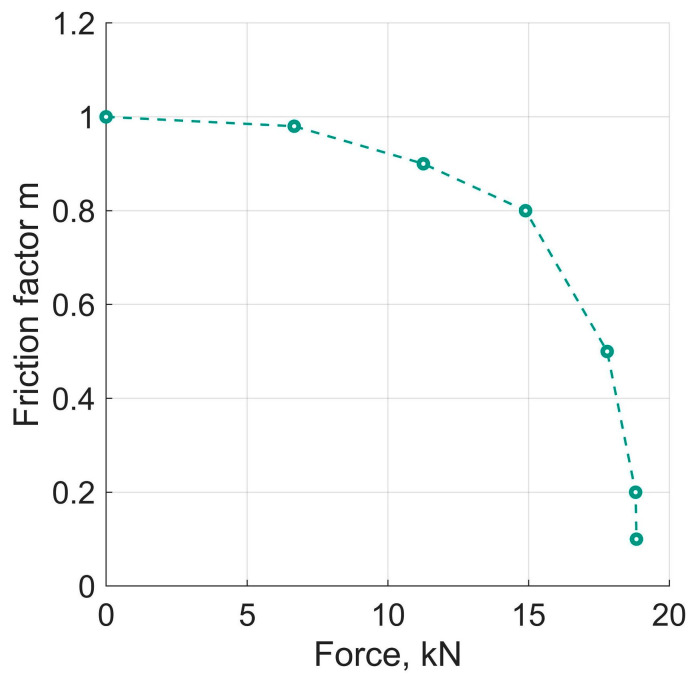
Dependence of the axial force ∆*P* at the onset of gripping on the friction factor m.

In the first set of numerical experiments, the force by which the anvils were pressed together was increased linearly until gripping and the onset of plastic deformation set in. Gripping, in the generalised sense, was determined by monitoring the decrease in the specimen thickness and the increase, from a zero level, at the geometrical centre of the specimen taken as a reference point. The magnitude of m and the corresponding value of ΔP at the moment of gripping were then plotted on the ordinate axis and the abscissa of a diagram (cf. Figure 6 below).

In the second set of numerical experiments the friction factor m was specified. The specimen was loaded by applying a certain force ΔP, followed by a rotation of the upper anvil. The quantities controlled in these computational experiments were ΔP (which dropped when the anvil rotation begun and then increased, reaching a prescribed level), torque, specimen thickness, the deformation rate, and the effective (equivalent) von Mises strain at the abovementioned reference point.

### 4.2. Results of Numerical Experiments

Numerical experiments of the first set revealed that for m<1, gripping of the specimen was associated with a decrease in its thickness and an increase in the diameter. A prerequisite for that is that the axial pressure exceeds the hydrostatic pressure. As mentioned in the foregoing section, this condition was captured in the magnitude of ΔP. For the case when m=1 held, plastic deformation occurred at ΔP=0, without any non-shear flows. The results of the first set of the numerical experiments are displayed in Figure 6. In essence, the curve in Figure 6 corresponds to the arch AD in Figure 3.

**Figure 7:**
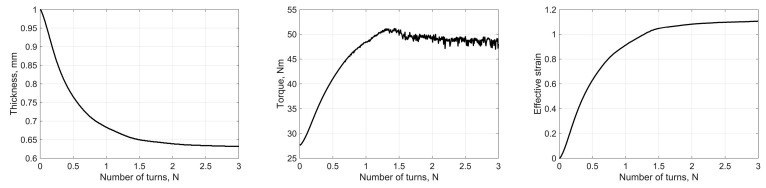
The results of the second set of numerical experiments for m = 0.9. The axial force was increased up to ΔP=15 kN and was fixed thereafter. Slippage of the entire specimen (without deformation) set in after approximately two full rotations.

Representative results of the second set of numerical experiments are shown in Figure 7. They confirm the conclusion made in the previous section: For m<1 and a fixed ΔP, the deformation of the specimen terminated when its slippage as a whole relative to the anvils began.

## 5. Discussion

We can now demonstrate that several facts known in the mechanics of HPT can be explained in terms of specimen gripping in a generalised sense, according to Equation (10), as opposed to Equation (1).

First of all, Equation (10) resolves the question posed in the introduction: How can HPT be realised when the specimen surface is covered with oxides or impurity-containing film preventing the friction force from attaining its limit value k? The answer is that in that case the insufficient magnitude of τ in Equation (4) is compensated for by the difference of the stress components $\left(\sigma_{zz}-\sigma_{rr}\right)$ associated with non-shear flows. The latter manifest themselves in the well-known effect of the spreading of the specimen material across the anvils and its extrusion into the gap between the anvils, away from the working zone.

A similar question relates to the HPT processing of stacks of layered materials [6,9] in the case when the constituent materials have different shear strength (e.g., alternating layers of hard and soft materials). According to Equation (1), plastic deformation of a hard layer is only possible if the friction stress at its boundaries can attain the level of the shear stress for the hard material $k_{h}.\;$ However, the shear stress at the boundaries of the soft layer cannot exceed its shear strength $k_{s}$. These requirements contradict each other, as these layers have a common boundary and must experience the same shear stress despite the inequality $k_{s}<k_{h}$. The contradiction is resolved by virtue of non-shear flows. In the case of layered materials, not only do non-shear flows result in spreading of the specimen, but they also lead to intrusion of the softer material into the harder layer [27].

Practical issues of estimating the effective (equivalent) strain realistically accumulated during HPT are caused by the specimen slippage, which can be regarded as a result of insufficient gripping in the sense of Equation (1). However, according to this condition, gripping cannot be “insufficiently good”: It either occurs, i.e., Equation (1) is fulfilled and no slippage takes place, or it is not fulfilled, and slippage does take place but no gripping takes place. Unlike Equation (1), Equation (10) opens up the possibility of slippage of the deforming material on the anvil surface. As discussed in Section 2, slippage reduces the accumulated effective (equivalent) strain. This is precisely what is found in the experiment in [28]. The magnitude of the highest attainable effective strain can be evaluated using Equation (37).

We saw in Section 1 that, for a fixed pressure, with increasing anvil rotation angle the HPT process approaches its feasibility limit (point B in Figure 5). At this boundary, the specimen starts slipping relative to the anvils, which blocks the growth of its equivalent strain. One may obtain the wrong impression that deformation of the specimen goes on, with no outflow of the material from the working zone of the anvils taking place. The numerical simulation results presented in Section 2 (see Figure 4 and the accompanying text) show that such a situation can be revealed when a higher pressure is used, which leads to renewed gripping enabling continued deformation of the specimen. In other words, increased axial pressure should raise the attainable equivalent strain of the specimen. This conclusion is in accordance with the results of real and numerical experiments [22,28].

As mentioned in the introduction, the tendency to slip becomes more pronounced with the growing hardness of the specimen material. This can be rationalised by considering the diagram in Figure 3 in non-dimensional coordinates $\left(P/K,\;M/K\right)$. Then, the abscissa of point C , representing the deformation regime, is given by $P_{c}/K$. All other conditions being the same, with increasing K this point moves to the left, closer to the boundary B of the HPT feasibility region, where plastic deformation of the specimen ceases. The closer to this boundary the system is, the higher is the probability of disruption of the gripping in the generalised sense, as the resource of the friction force becomes more and more limited.

It is to be expected that at point B the so called “stick–slip” effect may occur, which is common for frictional motion in systems where the shear stress for static friction $\tau_{st}$ (stick) and sliding friction $\tau_{sl}$ (slip) are different [29,30,31,32]. The former acts in the absence of macroscopic displacement of the contacting bodies, whereas the latter is at play when relative sliding sets in. A transition to relative sliding occurs when the stress τ at the contact surface attains the value of $\max\tau_{st}$, the highest possible level of the static friction. It is known from theories of friction that $\max\tau_{st}\mathrm{ecxceeds}\;\tau_{sl}$. For a system with mass and elasticity, this leads to the occurrence of oscillatory motion at the beginning of the gliding stage. The oscillations lead to cessation of gliding within a timeframe smaller than the half-period. After a certain amount of time of sticking, a new sliding episode follows, and these alternating events go on and on [29,30,31,32]. It is quite possible that at point *B* the slippage of the specimen on the anvils occurs according to this “stick–slip” scenario, rather than in a continuous way, since when deformation stops the flow of the material with respect to the anvils ceases instantaneously. This assumption is supported by the occurrence of audible creaking in the final stages of HPT deformation of hard materials—a common feature of “stick–slip”.

## 6. Conclusions

We have shown that viewing HPT just as a process of mere simple shear limits researchers in their understanding of the observed phenomena. A broader viewpoint on the mechanics of HPT, which admits the possibility of non-shear flows, enables a generalisation of the gripping condition. This facilitates a better control of these flows and makes it possible to achieve improved properties of HPT-processed materials.

The generalised Equation (10) provides a rational explanation to several observed effects. The currently accepted gripping criterion given by Equation (1) is a special case of Equation (10).

The following results obtained in this work should be highlighted because of their significance.

- In the (normal stress, shear stress) and (axial force, torque) coordinates, the generalised gripping condition of Equation (10) is represented by a “gripping arch,” distinct from a single point as a representation of the old gripping condition in the form of Equation (1). The upper boundary of the “gripping arch” is determined by the friction on the contact surface between the specimen and the anvils.
- The fulfilment of Equation (1), i.e., of the equality m=1, is the only possibility of plastic deformation of the HPT specimen by shear only. For m<1, shear under HPT is necessarily accompanied by non-shear flows. Unlike the former case, for m smaller than unity the deforming material always slips relative to the anvil surfaces.
- Furthermore, for m<1 gripping of the specimen in a generalised sense occurs when pressure exceeds a certain threshold, whose magnitude is greater the smaller m is.
- For m<1 the intensity of non-shear flows at a fixed axial decreases with the growing anvil rotation angle. At a certain moment plastic deformation of the specimen terminates and is followed by the slippage of the specimen as a whole relative to the anvils.
- The attainable level of the equivalent strain accumulated in the specimen gripped according to Equation (10) increases with an increase in the axial pressure. It is noteworthy that this equivalent strain always stays below the one calculated in terms of the conventional gripping criterion of Equation (1).

The plasticity locus for HPT proposed in this study opens the possibility to control the plastic flows involved and tune the formation of the microstructure and the properties of the processed material.

The article does not give final answers to all questions relating to gripping and non-shear flows. We are of the opinion, however, that the results presented provide a faithful description of several salient effects observed in the experiment. We trust that the generalised approach taken in this work offers some guidance to researchers and engineers for developing the HPT technology and defining new experiments.

## Data Availability

Not applicable.

## References

[1] Bridgman P.W. (1935). Effects of high shearing stress combined with high hydrostatic pressure. Phys. Rev..

[2] Bridgman P.W. (1938). Polymorphic Transitions up to 50,000 Kg/Cm of Several Organic Substances. Proc. Am. Acad. Arts Sci..

[3] Zhilyaev A.P., Langdon T.G. (2008). Using high-pressure torsion for metal processing: Fundamentals and applications. Prog. Mater. Sci..

[4] Valiev R.Z., Estrin Y., Horita Z., Langdon T.G., Zehetbauer M.J., Zhu Y.T. (2016). Producing bulk ultrafine-grained materials by severe plastic deformation: Ten years later. JOM.

[5] Estrin Y., Vinogradov A. (2013). Extreme grain refinement by severe plastic deformation: A wealth of challenging science. Acta Mater..

[6] Han J.K., Herndon T., Jang J.I., Langdon T.G., Kawasaki M. (2020). Synthesis of hybrid nanocrystalline alloys by mechanical bonding through high-pressure torsion. Adv. Eng. Mater..

[7] Bachmaier A., Pippan R. (2013). Generation of metallic nanocomposites by severe plastic deformation. Int. Mater. Rev..

[8] Krämer L., Champion Y., Pippan R. (2017). From powders to bulk metallic glass composites. Sci. Rep..

[9] Kawasaki M., Han J., Lee D., Jang J., Langdon T.G. (2018). Fabrication of nanocomposites through diffusion bonding under high-pressure torsion. J. Mater. Res..

[10] Kulagin R., Beygelzimer Y., Bachmaier A., Pippan R., Estrin Y. (2019). Benefits of pattern formation by severe plastic deformation. Appl. Mater. Today.

[11] Beygelzimer Y., Estrin Y., Kulagin R. (2015). Synthesis of hybrid materials by severe plastic deformation: A new paradigm of SPD processing. Adv. Eng. Mater..

[12] Edalati K., Horita Z., Langdon T.G. (2009). The significance of slippage in processing by high-pressure torsion. Scr. Mater.

[13] Gunderov D.V., Astanin V.V., Sharafutdinov A.V., Bhatt J. (2021). Slippage during high-pressure torsion processing of Vitreloy 105 bulk metallic glass. J. Phys. Conf. Ser..

[14] Gunderov D.V., Churakova A.A., Sharafutdinov A.V., Sitdikov V.D., Astanin V.V. (2022). Slippage during high-pressure torsion of commercially pure titanium and application of accumulative HPT to it. IOP Conf. Ser. Mater. Sci. Eng..

[15] Bridgman P.W. (1936). Shearing Phenomena at High Pressure of Possible Importance for Geology. J. Geol..

[16] Nielsen C.V., Bay N. (2018). Review of friction modeling in metal forming processes. J. Mater. Process. Technol..

[17] Levanov A.N. (1997). Improvement of metal forming processes by means of useful effects of plastic friction. J. Mater. Process. Technol..

[18] Lee D.J., Yoon E.Y., Lee S.H., Kim H.S. (2012). Finite element analysis for compression behavior of high pressure torsion processing. Rev. Adv. Mater. Sci..

[19] Kamrani M., Levitas V.I., Feng B. (2017). FEM simulation of large deformation of copper in the quasi-constrain high-pressure-torsion setup. Mater. Sci. Eng. A.

[20] Beygelzimer Y., Kulagin R., Toth L.S., Ivanisenko Y. (2016). The self-similarity theory of high pressure torsion. Beilstein. J. Nanotechnol..

[21] Pereira P.H.R., Figueiredo R.B., Cetlin P.R., Langdon T.G. (2014). Using finite element modelling to examine the flow process and temperature evolution in HPT under different constraining conditions. IOP Conf. Ser. Mater. Sci. Eng..

[22] Song Y., Wang W., Gao D., Yoon E.Y., Lee D.J., Kim H.S. (2014). Finite element analysis of the effect of friction in high pressure torsion. Met. Mater. Int..

[23] Kachanov L.M. (2004). Fundamentals of the Theory of Plasticity.

[24] Prager W., Hodge P.G. (1951). Theory of Perfectly Plastic Solids.

[25] Rabotnov Y.N. (1979). Mechanics of Deformable Solids.

[26] QForm-3D. https://www.qform3d.com/.

[27] Tavakkoli V., Mazilkin A., Scherer T., Mail M., Beygelzimer Y., Baretzky B., Estrin Y., Kulagin R. (2021). Instability of a Molybdenum Layer under Deformation of a CuMoCu Laminate by High-Pressure Torsion. Mater. Lett..

[28] Edalati K., Matsubara E., Horita Z. (2009). Processing Pure Ti by High-Pressure Torsion in Wide Ranges of Pressures and Strain. Metall. Mater. Trans. A.

[29] Ishlinsky A.Y., Kragelsky I.V. (1944). On jumps under friction. J. Tech. Phys..

[30] Bishop R.E.D. (1965). Vibration.

[31] Persson B.N.J. (1998). Sliding Friction, Physical Principles and Applications.

[32] Johnson K.L. (1987). Contact Mechanics.

